# Brief report of protective factors associated with family and parental well-being during the COVID-19 pandemic in an outpatient child and adolescent psychiatric clinic

**DOI:** 10.3389/fpsyg.2022.883955

**Published:** 2022-09-12

**Authors:** Tamaki Hosoda Urban, Deborah Friedman, Maysa Marwan Kaskas, Alessandra J. Caruso, Katia M. Canenguez, Nancy Rotter, Janet Wozniak, Archana Basu

**Affiliations:** Department of Psychiatry, Massachusetts General Hospital and Harvard Medical School, Boston, MA, United States

**Keywords:** parental wellbeing, child and adolescent mental health, resilience, family functioning, COVID-19 pandemic, social support, protective factors, cognitive behavioral tools

## Abstract

Families of children with mental health challenges may have been particularly vulnerable to emotional distress during the COVID-19 pandemic. This cross-sectional study surveyed 81 parents of children ages 6–17 years receiving mental health treatment in an outpatient clinic during the pandemic. We sought to characterize the impact of the pandemic on family relationships and parental well-being. Additionally, regression and ANCOVA models examined associations between four potentially protective factors—parents’ psychological resilience, perceived social support, positive family experiences during the pandemic, and children’s use of cognitive or behavioral coping strategies—with family relationships and parental well-being. Findings suggest that families of children with mental health conditions experienced remarkable challenges to family relationships, parental well-being, and parents’ perceived capacity to support their children’s mental health. Nearly 80% of parents reported a negative impact of the pandemic on their own well-being, and 60% reported reduced ability to support their children’s mental health. Simultaneously, protective factors appeared to mitigate the negative impact of the pandemic. Particularly, support within the family (e.g., co-parenting) and from external sources (e.g., mental health services) were associated with better self-reported well-being for parents and their capacity to support their children. Children’s use of coping tools, likely enhanced by mental health treatment, was also positively related to better family relationships and parental ability to support children with mental health challenges. Our findings highlight the need for enhancing supports for families at multiple levels including individual skill-building, family-based/parenting support, and community-based support.

## Introduction

For children and their parents, the COVID-19 pandemic has been a complex stressor. Increased isolation due to “social distancing” and school closures, reduced access to supports (e.g., after-school programs and peer interactions), potential changes to parents’ employment and household demands, personal or communal losses and grief, and the ongoing viral disease threat have all contributed to increased distress and mental health concerns. Indeed, several studies have noted a broad-spectrum increase in mental health (Viner et al., 2022), developmental (Racine et al., 2022), and school-related challenges for children (Engzell et al., 2021; Dudovitz et al., 2022), as well as negative effects on families (Marchetti et al., 2020; Spinelli et al., 2020; Calvano et al., 2021; Rothe et al., 2021) during the pandemic. Available studies also suggest that the pandemic has resulted in increased familial conflicts (Cassinat et al., 2021) and negatively affected parental well-being (Patrick et al., 2020).

Individuals with pre-existing mental health conditions experienced more mental health challenges in the pandemic (Neelam et al., 2021). A likely increase in children’s mental health needs, in addition to the elevated demands on parents, and a negative impact on parents’ own emotional distress (American Psychological Association, 2021a), may collectively amplify stress in parent–child relationships. However, protective factors, which may include individual factors (e.g., child’s use of coping skills and parental psychological resilience; Benson, 2010; Cappa et al., 2011), family-level factors (e.g., positive family experiences), and social support can mitigate the negative effect of such stressors.

Promoting the use of a range of cognitive (e.g., practicing gratitude) and behavioral (e.g., physical activity) coping tools is not only a key focus for most mental health treatments, but has also been endorsed as part of public health recommendations for effective coping during the pandemic for people of all ages (American Psychological Association, 2021b). Additionally, parents’ own psychological resilience, defined as the general capacity for positive adaptation in adversity (Connor and Davidson, 2003), is also associated with more adaptive or attuned parenting (Gavidia-Payne et al., 2015; Cousineau et al., 2019). Adaptive parenting in turn may support children’s effective coping during stressful experiences. Similarly, studies conducted prior to the pandemic show that positive family time is associated with numerous benefits for family members and more positive parent–child relationships (Buswell et al., 2012; Hodge et al., 2015). More recently, in a national survey of parents, one-third of families reported that they experienced more positive time together during the pandemic (DeAngelis, 2021). Yet the potential moderating impact of such positive family experiences has not been examined in the context of pandemic-related stressors. Finally, social support, including emotional, financial, or practical support from others outside the family or household, is one of the most robust protective factors associated with family resilience (McCubbin and McCubbin, 1996).

Despite available evidence that protective factors can buffer the impact of stressors, few studies have examined these factors when considering pandemic-related stressors, and families of children with mental health challenges remain particularly under-examined. Accordingly, we sought to characterize the impact of the pandemic on family relationships and parental well-being in families of children receiving outpatient mental health care. We also examined associations between these indicators of family and parent functioning and four potentially protective factors: parental psychological resilience, perceived social support, positive family experiences during the pandemic, and children’s use of coping tools. Investigating such protective factors can inform future resilience-promoting research and interventions for families.

## Materials and methods

### Study design and sample

This study was conducted at an outpatient child and adolescent psychiatric clinic in an academic medical center in an urban city, in the Northeast U.S. region, from March 2021 through June 2021. For the purposes of our study, the term “parents” is used to include biological or adoptive parents, or legal guardians (i.e., the child’s primary legal caregiver per the medical records). Parents of children receiving mental health care were invited to complete a cross-sectional online survey about their experiences during the pandemic. Inclusion criteria were: (1) parents of children ages 0–17 years; (2) parents of children who received mental health care in the clinic between March 2019 (within 1 year prior to the pandemic) and February 2021; (3) parents who could complete the survey in English; and (4) consistent with hospital policies, only parents who had previously consented to being contacted for research studies. A recruitment letter was sent via a secured hospital-based electronic communication system to eligible parents. Parents who were not enrolled in the hospital’s system electronic communication system were invited to participate by phone or informed of the study during clinical visits. This study was approved by the hospital’s Institutional Review Board.

### Measures

#### Child and family characteristics

Parents reported on child demographics (e.g., age, race/ethnicity, and sex assigned at birth) and family characteristics (e.g., financial concerns). Type of health insurance and the most recent child mental health diagnoses (based on a psychologist or psychiatrist’s evaluation as part of providing mental health care) were extracted from the child’s electronic medical record to characterize the sample once survey data were collected.

#### Family relationship functioning and parental well-being

*Impact on relationships and parental well-being* were assessed through a brief survey created for this study. Parents were asked about how the pandemic affected the following relationships and their own well-being: (1) overall relationships within family/household; (2) with extended family/friends; (3) community members (e.g., co-workers and neighbors); (4) parents’ overall ability to support their children with behavioral or mental health conditions; and (5) parents’ own emotional well-being. Response choices on five-point Likert scale included “very negatively affected,” “somewhat negatively affected,” “unaffected,” “somewhat improved,” “improved a lot,” and a “does not apply” option (e.g., if there is no co-parent or partner). A mean score for each item was created for each respondent.

#### Protective factors

*Parent’s psychological resilience* was measured using the standardized 10-item Conner-Davidson Resilience Scale (CD-RISC-10; Connor and Davidson, 2003). The CD-RISC-10 has been widely used to assess psychological resilience (e.g., adapting to changes, seeing a humorous side of issues, and believing in own ability to achieve a goal). This measure has robust psychometric properties (Cosco et al., 2016). Response options used a five-point Likert scale ranging between “not very true” to “true nearly all the time.” Standard scoring was used to create a sum score, which can range from 0 to 40, for each respondent.

*Perceived social support* was measured using a survey adapted from the validated Duke Social Support and Stress Scale (Parkerson et al., 1991). Parents were asked about how supported they felt by their spouse/partner/significant other, child(ren), parents, extended family, friends, neighbors, co-workers, or another source of support (including mental health services) during the pandemic. Response options used a five-point Likert scale ranging between “not at all supported” to “extremely supported.” A mean score for the following four categories of support was created for each respondent: (1) overall support (all items); (2) immediate family (spouse/partner/significant other, child); (3) extended family (extended family, parent); and (4) external support (friends, neighbors, co-workers, and another source of support).

*Positive family experiences during the pandemic* were assessed through a survey created for this study. Parents were asked about the following eight types of experiences: enjoying time together, improved communication with child’s school, gaining more insight into child’s schoolwork or learning, being more productive working from home, less commute-related stress, able to focus more on pleasant or meaningful activities, less exposure to stressful situations than usual (e.g., work setting), and improved connection with friends or community members. Response options used a three-point Likert scale ranging from “not at all” to “very much so.” A total sum score, which can range from 0 to 16, was created for each respondent.

*Child’s use of psychological and behavioral coping tools* was assessed through a survey created for this study. Parents were asked about how often their child utilized 17 possible coping strategies during the pandemic, including: prioritizing sleep or physical activity, getting time outdoors, staying socially connected, having regular family conversations, practicing mindfulness, and connecting with professional mental health services. Responses options ranged between “not at all” to “a great deal” on a three-point Likert scale. A total sum score, which can range from 0 to 68, was created for each respondent.

### Statistical analysis

Regression and ANCOVA models examined associations between impact on relationships and parental well-being with four protective factors (parental psychological resilience, perceived social support, positive family experiences during the pandemic, and child’s use of coping tools), respectively. Covariates included child’s age (continuous), race/ethnicity (non-Hispanic White *vs*. others), sex at birth (female vs. male), and financial concerns (yes vs. no). The significance threshold for all analyses was *p* = 0.05.

## Results

During the study period, 1,220 parents were invited to participate. Only 96 parents responded to the survey. After excluding respondents with duplicate records or those who did not complete the majority of the survey, data from 81 parent respondents were available for analyses. Table 1 presents child demographics and family characteristics. The average child age was 13.4 years (SD = 3.1) with 61.7% between 12 and 17 years. Most children were female at birth (56.8%) and cisgender (93.8%: 6.2% were gender expansive). Children were identified as gender expansive if their gender identity as reported by their parents was listed as transgender, non-binary, genderqueer, or other gender diverse. Most children identified as non-Hispanic White (65.4%). The most prevalent mental health diagnosis among children was an anxiety disorder (64.2%), followed by ADHD (44.0%). Among parents, 77% were married/living together or co-parenting; 13.6% reported financial concerns over essential expenses during the pandemic. More than two-thirds of families had private health insurance.

**Table 1:** Child demographics and family characteristics (*N* = 81).

	*n*	%
Child Age (mean, SD)	13.4 (3.1)	
Ages 0–5	0	0.00
Ages 6–11	31	38.27
Ages 12+	50	61.73
Child Sex		
Female	46	56.79
Male	35	43.21
Child gender identity		
Gender Expansive	5	6.17
Cisgender	76	93.83
Child race/Ethnicity		
Hispanic	10	12.35
Non-Hispanic White	53	65.43
Non-Hispanic Black or AA	1	1.23
Non-Hispanic Asian or Pacific Islander	1	1.23
Non-Hispanic Native or Indigenous American	1	1.23
Non-Hispanic Biracial or multiracial	11	13.58
Missing	4	4.94
Child mental health diagnosis		
Mood disorder	35	43.21
Anxiety disorder	52	64.20
Adjustment disorder	8	9.87
ADHD	40	43.98
Other diagnosis (e.g., eating disorder, PTSD, elimination disorder, and gender dysphoria)	58	71.60
Parent gender identity		
Male	8	9.88
Female	73	90.12
Relationship to child		
Parent	78	96.30
Legal guardian	0	0.00
Missing	3	3.70
Parenting arrangement		
Single parent	6	7.41
Living together (e.g., married)	59	72.84
Divorced or separated	11	13.58
Co-parenting	3	3.70
Not co-parenting	1	1.23
Other	1	1.23
Insurance type		
Public	13	16.05
Private	68	83.95
Financial Concerns		
Were you concerned about your ability to afford essential expenses (e.g., food or medicine)? – YES	11	13.58

### Impact of the pandemic on parental and family relational functioning

Table 2 presents data on parental experiences of the pandemic. Negative impacts on family/household relationships were reported by 45.5%; 50.6% reported negative impacts on extended family/friend relationships and 35.3% on relationships with community members. Negative impacts were reported by 60.0% related to parental ability to support their child with mental health challenges and 79.5% related to their own well-being. Parents reported they felt most supported by their spouses/partners/significant others and least supported by their neighbors during the pandemic. Most parents (60.8%) reported their individual psychological resilience to be poor (i.e., the lowest quartile), whereas 10.8% reported a high level of psychological resilience (i.e., the highest quartile).

**Table 2:** Parent/guardian experience during the COVID-19 pandemic.

	*n*	% (valid)	Mean	SD
The impact of pandemic on…				
Overall relationships within your family/household				
Negatively affected (somewhat—very)	35	45.45		
Unaffected	19	24.68		
Improved (somewhat—a lot)	23	29.87		
Overall relationships with extended family/ friends				
Negatively affected (somewhat—very)	40	50.63		
Unaffected	31	39.24		
Improved (somewhat—a lot)	8	10.13		
Overall relationships with co-workers or other people at work				
Negatively affected (somewhat—very)	23	35.33		
Unaffected	36	52.17		
Improved (somewhat—a lot)	10	14.49		
Overall relationships with neighbors or community members				
Negatively affected (somewhat—very)	27	35.53		
Unaffected	39	51.32		
Improved (somewhat—a lot)	10	13.16		
Your overall ability to support your child with a mental or behavioral health condition				
Negatively affected (somewhat—very)	42	60.00		
Unaffected	19	27.14		
Improved (somewhat—a lot)	9	12.86		
Your own overall emotional well-being (such as anxiety or mood)				
Negatively affected (somewhat—very)	62	79.49		
Unaffected	14	17.95		
Improved (somewhat—a lot)	2	2.56		
Parental psychological resilience (CD-RISC-10)			25.95	10.03
Quartile				
Lowest	45	60.81		
Second	9	12.16		
Third	12	16.21		
Highest	8	10.81		
How supported did you feel by the following? (ranged from “0=not at all” to “4=extremely”)				
Spouse, partner, significant other			2.46	1.40
Child(ren)			2.07	1.18
Parents			2.23	1.28
Extended family			1.88	1.38
Friends			2.25	1.21
Neighbors			1.58	1.33
Coworkers			2.16	1.27
Other source of support			2.09	1.54
Positive aspects to family's experience (0=not at all, 1=somewhat, 2=very much so)				
Your family enjoyed time together that you would otherwise not have had			1.48	0.62
Communication with your child(ren)'s teacher or school improved			0.82	0.69
You gained more information or insight into your child(ren)'s schoolwork or learning style			1.18	0.72
You were able to work well and be productive from home			1.13	0.69
Your commute-related stress was reduced or eliminated			1.60	0.65
You were able to focus more on other pleasant or meaningful activities (such as physical activity or hobbies)			0.96	0.77
You had less exposure to stressful situations or relationships than usual (such as at work or in other settings)			0.94	0.85
Your connections with your friends, neighbors or other community members improved			0.59	0.68
Child’s use of psychological and behavioral coping tools (ranged from “0=not at all” to “4=extremely”)			1.28	0.97
Focused on getting good sleep			1.43	1.00
Was physically active (such as walking, running, yoga, or other activities)				
Made an effort to eat healthy			1.52	1.01
Remained socially connected through video chats, phone calls, etc.			2.04	1.10
Had regular family conversations to check-in with each other (all together or with a parent or sibling)			2.01	0.96
Used strategies to stay organized (such as a family calendar, organizational app, or planned breaks)			1.45	1.09
Practiced gratitude (recognizing something they were grateful for)			1.22	1.03
Did something helpful for someone else			1.49	0.92
Took regular breaks from social media and/or the news			1.14	0.90
Took part in religious or spiritual practices/ prayed			0.66	0.98
Practiced mindfulness-based activities (such as self-compassion or meditation)			0.70	0.92
Practiced behavioral relaxation (such as deep breathing)			1.01	0.97
Processed their feelings through journaling/ writing			0.71	0.98
Focused on a hobby			1.71	0.98
Spent time outdoors/in nature			1.74	0.95
Connected with a mental health professional			2.01	1.01
Another activity to maintain or improve their well-being			1.25	1.17

### Impact of protective factors on parental well-being and family relational functioning

Table 3 presents associations between protective factors with families’ relational functioning and parental well-being. The overall pattern of results did not change when adjusting for child’s age, race/ethnicity, sex at birth, and financial insecurity. Therefore, results in adjusted models are presented below.

**Table 3:** The associations of protective factors with families’ relational functioning and parental well-being in adjusted models.

	Relationships within the family	Relationships with extended family/friends	Relationships with community members	Ability to care for child with mental health condition	Well-being
	*MR^2^*	*MR^2^*	*MR^2^*	*MR^2^*	*MR^2^*
Parental resilience	0.010	0.119^*^	0.031	0.124	0.075
Social support					
Overall support	0.064	0.074	0.035	0.235^**^	0.167^**^
Immediate family	0.155^**^	0.060	0.021	0.314^***^	0.162^**^
Extended family	0.023	0.110	0.020	0.128	0.205^***^
External support	0.036	0.065	0.060	0.217^**^	0.111^*^
Positive family experiences	0.108^**^	0.131^*^	0.465	0.176^*^	0.187^***^
Child’s use of coping tools	0.135^**^	0.112	0.062	0.180^*^	0.046

* *p* < 0.05;

** *p* < 0.01;

*** *p* < 0.001.

Models are adjusted for child’s age, race/ethnicity, sex at birth, and financial insecurity.

Parents with greater psychological resilience reported a greater positive impact of the pandemic on relationships with their extended family/friends (*MR*^2^ = 0.119, *p* = 0.046). There was no significant association between parental psychological resilience and the impact on other types of family relationships (e.g., immediate family and community relationships) or on self-reported parental well-being.

Perceived social support (i.e., overall, immediate family, and external) during the pandemic was associated with improved parental ability to support the child with mental health challenges and parental well-being. Specifically, perceived support from extended family during the pandemic was associated with improved parental well-being (*MR*^2^ = 0.205, *p* < 0.001). Perceived support within the immediate family was associated with improved family relationships (*MR*^2^ = 0.155, *p* = 0.002). As examples of each type of support, some parents reported “co-parenting” for immediate family support, “child’s grandparents” as extended family support, and “mental health services” as external support in the survey.

Higher ratings of positive family experiences during the pandemic were associated with improved relationships within the immediate family (*MR*^2^ = 0.108, *p* = 0.007) and with extended family (*MR*^2^ = 0.131, *p* = 0.027). Positive family experiences were also associated with self-reported parental ability to care for their child with mental health challenges (*MR*^2^ = 0.176, *p* = 0.034), and parental well-being (*MR*^2^ = 0.187, *p* < 0.001) during the pandemic. Finally, parents who identified their child as using more coping tools reported improvements in family relationships (*MR*^2^ = 0.135, *p* = 0.002) and parental ability to care for their child with mental health challenges (*MR*^2^ = 0.180, *p* = 0.028) during the pandemic.

## Discussion

This study identified significant negative impacts of the pandemic on family relationships and parental well-being. Simultaneously, findings also suggest that higher levels of protective factors, including parental resilience, perceived social support, positive family experiences, and children’s use of cognitive or behavioral coping strategies, were associated with lower negative impacts on families and parents. This study is novel in its exploration of these factors in a sample of outpatient treatment-engaged children and families.

### Family relationship functioning and parental well-being

Study findings suggest that families of children with mental health needs experienced significant challenges during the pandemic. Particularly, parents reported negative impacts across all types of relationships, including within their own family or household (45%), with extended family and friends (51%), and others (e.g., co-workers/neighbors; approximately 35%). A majority of parents also noted negative effects on their ability to support their children’s mental health needs (60%), and their own well-being (79%). Additionally, nearly two-thirds of parents reported their psychological resilience (e.g., adaptability and self-efficacy) to be in the lowest quartile. Psychological resilience assessed in this study measured parental general resilience. However, it is possible the pandemic negatively impacted parents’ perception of their own psychological resilience. Parental well-being can impact child mental health (Amrock and Weitzman, 2014), and available data from during the COVID-19 pandemic suggest a similar pattern of associations (Verger et al., 2021). As such, our findings highlight possible needs for family-centered interventions for children with mental health conditions, in addition to child-focused individual interventions.

Several factors were associated with reduced negative impact of the pandemic for parents and families. First, parents with higher levels of self-reported psychological resilience were less likely to report a negative impact of the pandemic on their relationships with extended family/friends. This suggest that parents with greater psychological resilience may maintain close connections with loved ones outside their household, which could have contributed to reduced feelings of isolation or increased perceived social support, during the pandemic.

Unexpectedly, we did not find association between parental psychological resilience and their self-reported well-being. We speculate that different factors may have affected parental well-being across the spectrum of psychological resilience. For some families, including potentially parents with lower psychological resilience, other protective factors (e.g., social support) may have served as a buffer. Whereas for other parents, even potentially those with higher individual resilience, pandemic stressors may still have had a negative impact on their well-being. Thus, this unexpected finding may represent complex interactions between multiple stressors and protective factors at different levels.

Parents who perceived greater social support reported better individual well-being and better ability to support their child’s mental health needs in the pandemic. This is consistent with the well-established research prior to the pandemic (McCubbin and McCubbin, 1996) but also available data from the pandemic (Wu et al., 2020). These findings underscore the importance of promoting families’ access to social support, including identifying barriers to accessing adequate support during crises.

The protective effects of positive family experiences during the pandemic (e.g., enjoying more quality time together) were also demonstrated across domains of family functioning and parental well-being. In our sample, most participants identified as non-Hispanic White, with private insurance and reported limited financial stress. There are robust data that members of minoritized communities and families from under resourced communities were disproportionately affected by the pandemic, including mental health consequences (Mackey et al., 2021; Thomeer et al., 2022), which is not represented in our study. However, it is also possible that families of color who have cultural values that involve closer family or community relationships or share childcare with extended family in the same household (e.g., inter-generational homes or families; Taylor et al., 2013) may have had support systems that buffered the impact of stressors on these families or, even on parent’s own well-being (as per our aforementioned findings). Thus, positive family experiences may reflect various contextual factors such as parental ability to work remotely, economic resources, or other extended family/communal relationships that facilitate more positive experiences for families. It is essential to understand these diverse contextual factors across families from various racial/ethnic groups when considering interventions for families.

Parents who indicated that their child used more cognitive or behavioral coping tools during the pandemic also noted less negative effects on their own individual well-being, better ability to support their child, and better overall family relationships. We surveyed parents on their child’s use of a broad range of coping skills previously shown to be associated with improved social and emotional functioning, and are a typical part of most mental health treatments. Our findings add to the literature that adaptive child coping has positive effects on family functioning and parental well-being, and are consistent with recent calls for implementing similar interventions in other settings (e.g., schools) to address the pandemic’s impact on children (Varghese and Natsuaki, 2021).

Taken together, our findings highlight the importance of enhanced support for parents of children with mental health conditions and prolonged stress. Clinically, these results underscore the importance of acknowledging the needs of parents with children with mental health challenges and point to the potential benefits of increasing access to treatment not only for children but also for their parents as well. Our findings suggest the need to bolster protective factors for families of children with mental health challenges at multiple levels, including individual skill-building, family-centered/parenting support, and community-based support (Stark et al., 2020).

### Limitations

Study findings should be interpreted in the context of some key limitations. First, the small study sample limits generalizability of our findings. Although the survey was available to parents in online and written formats, the response rate was low and may reflect a self-selection in respondents. Second, study respondents were largely (61.7%) parents of adolescents (ages 12–17 years), an age group that may have faced different challenges during the pandemic as compared to younger children (e.g., greater need for peer interaction or greater access to social media). However, the small sample size limits the possibility of additional stratified or sensitivity analyses. Third, our study did not collect data on parental mental health. Parents with their own mental health challenges may have experienced more relationship challenges or may have been more likely to report reduced individual well-being, and reduced ability to support their child. Fourth, the survey in this study was provided in English, thus limiting the ability to potentially reach all families with children receiving care in our clinic. Finally, this was a cross-sectional survey with data collected from March 2021 through June 2021. The pandemic experience has evolved over time. For example, there were changes to the availability of vaccines, surge and decline in variant-related COVID-19 infection rates, changes in schooling format (e.g., hybrid, online, and in-person), and travel restrictions. All these factors likely affected familial well-being. Hence, longitudinal studies are necessary to understand the long-term impact of the pandemic on children and parents’ functioning.

### Conclusion

During the pandemic, families of children with mental health conditions likely experienced increased challenges in maintaining healthy family relationships, parental well-being, and negative effects on parents’ capacity to support their children’s mental health. Simultaneously, protective factors were associated with reduced negative impact of the pandemic. Particularly, support within the family (e.g., co-parenting) and from external sources (e.g., mental health services) was associated with reduced negative effects on parental well-being and parents’ support of their children. Children’s use of coping tools, likely enhanced by mental health treatment, was also positive for family relationships. These findings highlight the need for supports for families at multiple levels, including social emotional skill-building, family-centered or parenting support, and interventions that promote social supports for families.

## Data availability statement

The datasets for this study cannot be made publicly available per the hospital review board policies. Requests to access the datasets should be directed to thosoda1@mgh.harvard.edu.

## Ethics statement

The studies involving human participants were reviewed and approved by Massachusetts General Hospital. Written informed consent from the participant’s legal guardian/next of kin was not required to participate in this study in accordance with the national legislation and the institutional requirements.

## Author contributions

TU: conceptualization, methodology, patient recruitment, data management, formal analyses, writing—original draft preparation, reviewing, editing, patient recruitment, and formatting. DF: conceptualization, methodology, patient recruitment, reviewing, and editing. MK: conceptualization, methodology, patient recruitment, and writing—original draft preparation. AC: data entry/management, formatting, reviewing, and editing. KC: patient recruitment and writing—original draft preparation. NR and JW: reviewing and editing. AB: conceptualization, methodology, patient recruitment, writing—original draft preparation, reviewing, and editing. All authors contributed to the article and approved the submitted version.

## Funding

We would like to thank Karestan Koenen, Department of Psychiatry, Massachusetts General Hospital, for intramural funding support.

## Conflict of interest

The authors declare that the research was conducted in the absence of any commercial or financial relationships that could be construed as a potential conflict of interest.

## Publisher’s note

All claims expressed in this article are solely those of the authors and do not necessarily represent those of their affiliated organizations, or those of the publisher, the editors and the reviewers. Any product that may be evaluated in this article, or claim that may be made by its manufacturer, is not guaranteed or endorsed by the publisher.
